# Host-directed therapeutic strategies against apicomplexan parasites: targeting purinergic P2 receptors

**DOI:** 10.1007/s11302-026-10145-7

**Published:** 2026-03-21

**Authors:** Mariana M. Chaves, Nayara Carvalho-Barbosa, Luiz Eduardo Baggio Savio, Robson Coutinho-Silva

**Affiliations:** 1 Institute of Biophysics Carlos Chagas Filho (IBCCF), Federal University of Rio de Janeiro (UFRJ), Rio de Janeiro, RJ, Brazil; 2 Edifício Do Centro de Ciências da Saúde, Bloco G. Av. Carlos Chagas Filho, 373. Cidade Universitária, Ilha do Fundão, Rio de Janeiro, RJ 21941-902 Brazil

**Keywords:** P2 receptors, Apicomplexan parasites, Toxoplasmosis, Malaria, Therapeutic targets

## Abstract

Apicomplexan parasites establish intracellular infections that profoundly alter host cell physiology and elicit complex immune responses. The long-standing coevolution between these parasites and vertebrate hosts has resulted in extensive overlap between parasite and host metabolic pathways, limiting the feasibility of conventional parasite-centered therapeutic approaches. Increasing evidence indicates that host-derived signals generated during infection play a decisive role in shaping parasite survival and dissemination. Among these signals, extracellular nucleotides released in response to cellular stress and tissue damage have emerged as key modulators of innate immune responses. These molecules are sensed by purinergic P2 receptors, which integrate danger signals with inflammatory and microbicidal pathways. This review examines how purinergic signaling contributes to host–parasite interactions during apicomplexan infections, with particular emphasis on *Toxoplasma gondii* and *Plasmodium* spp. We discuss the dual role of P2 receptors in coordinating immune responses and directly affecting parasite viability, highlighting their potential as targets for host-directed therapeutic strategies.

## Introduction

Parasitism within the phylum Apicomplexa is characterized by unicellular eukaryotic organisms that depend entirely on an intracellular lifestyle. While thousands of species have been formally described, estimates suggest that the real diversity of this group is vastly higher [1]. Several apicomplexan parasites have a profound impact on global health and agriculture, as they are responsible for debilitating infections in humans and livestock, affecting populations on a massive scale each year [2, 3]. These burdens underscore the importance of investigating fundamental aspects of apicomplexan biology, particularly mechanisms that enable host invasion, intracellular survival, and immune modulation. Moreover, as eukaryotic pathogens that replicate inside host cells, apicomplexans display biological strategies that are fundamentally distinct from those of bacteria and viruses. This combination of medical relevance and unique cellular organization makes Apicomplexa a compelling subject of study [4].

Apicomplexan parasites establish infections across a broad range of vertebrate hosts, including aquatic and terrestrial species, reflecting a prolonged and intimate evolutionary relationship with their hosts [5]. This extended coadaptation has imposed significant constraints on therapeutic development, as many biological processes essential for parasite survival rely on molecular pathways that closely resemble those of the host. As a consequence, pharmacological strategies aimed at disrupting parasite metabolism frequently risk collateral damage to host cells [6]. Understanding the mechanisms underlying protective immunity against apicomplexan parasites remains experimentally constrained, largely because experimental investigation in definitive hosts, such as humans and cattle, is not feasible. As a result, much of the current knowledge has emerged from studies in genetically tractable murine models [7]. During infection, intracellular replication of apicomplexan parasites typically culminates in host cell rupture and parasite dissemination. Control of parasite growth is strongly influenced by innate immune responses, particularly through effector molecules released by immune cells that restrict infectivity and limit parasite expansion [8–10]. Although the precise molecular targets of these responses remain incompletely defined, evidence indicates that parasite inhibition relies on coordinated microbicidal pathways.

Within this context of host cell disruption and innate immune control, signals released as a consequence of cellular stress and lysis have gained increasing attention as key regulators of antiparasitic immunity [11, 12]. Among these signals, extracellular nucleotides such as ATP emerge as conserved indicators of tissue damage and infection, rapidly accumulating in the extracellular milieu following membrane rupture or active release from stressed cells [13]. These nucleotides act as endogenous danger signals that are sensed by purinergic P2 receptors expressed on both immune and non-immune cells, thereby linking parasite-induced cellular damage to coordinated immune activation [14, 15]. By translating extracellular nucleotide accumulation into inflammatory, microbicidal, and regulatory responses, P2 receptors represent a molecular interface through which innate immune mechanisms can influence parasite survival and dissemination, even in the absence of direct parasite-specific recognition.

## P2 receptors

Parasitic infection profoundly alters the intracellular landscape of host cells, imposing metabolic constraints, ionic disequilibrium, and membrane remodeling that collectively reshape the local immune environment [16, 17]. These perturbations generate secondary signals that extend beyond the infected cell and influence surrounding tissues [18, 19]. Among these signals, extracellular nucleotides have emerged as critical mediators linking cellular stress to antimicrobial defense mechanisms and parasite control.

Rather than being constitutively available in the extracellular compartment, nucleotides such as ATP and UTP are released primarily as a consequence of pathological processes [20]. Infection-associated stressors, including hypoxia, exposure to cytotoxic mediators, and mechanical or metabolic damage to cellular membranes, trigger nucleotide efflux from viable cells through a combination of regulated export mechanisms and passive leakage [21, 22]. In inflamed tissues, this process is further intensified by widespread membrane disruption, leading to sustained nucleotide accumulation in the extracellular space [21]. This accumulation represents a decisive molecular event capable of shaping immune activation and directly influencing parasite viability.

The magnitude and duration of extracellular nucleotide signaling are tightly controlled by membrane-associated conduits, including pannexin channels, which modulate ATP availability under diverse physiological and pathological contexts [23]. By regulating the extracellular nucleotide pool, these channels indirectly determine the strength and spatial distribution of purinergic signaling perceived by immune cells and intracellular pathogens. Once present outside the cell, nucleotides function as endogenous alarm signals, engaging purinergic P2 receptors broadly expressed across immune and non-immune cell populations [14].

Purinergic receptors translate extracellular nucleotide cues into intracellular responses through two fundamentally distinct but complementary signaling strategies. The P2X receptor family comprises ATP-gated ion channels that rapidly alter membrane permeability and intracellular ionic homeostasis, whereas P2Y receptors signal through G protein–dependent pathways that reprogram cellular behavior. Seven P2X receptor subtypes (P2X1–P2X7) have been identified in humans, each displaying distinct biophysical properties, tissue distribution, and functional roles in inflammation and host defense [24, 25]. Collectively, P2X receptors mediate rapid calcium influx, potassium efflux, and changes in membrane potential that can profoundly affect both immune cell activation and the intracellular niches exploited by parasites.

Within this family, P2X7 has attracted particular attention due to its unique ability to transition from a small-conductance cation channel to a large pore upon sustained ATP stimulation [26, 27]. However, other P2X receptors also contribute to antimicrobial immunity by regulating leukocyte activation, cytokine release, chemotaxis, and phagocytic function [28–31]. Through these coordinated actions, P2X receptors act as immediate sensors of extracellular nucleotide accumulation and as early amplifiers of host responses to infection.

Activation of P2X receptors initiates a spectrum of downstream effects that collectively create hostile intracellular environments for parasites. These responses include inflammasome activation, secretion of pro-inflammatory cytokines such as IL-1β and IL-18, release of extracellular vesicles, and production of inflammatory lipid mediators, including leukotriene B₄ [20, 28, 32, 33]. In parallel, P2X receptor signaling enhances classical microbicidal mechanisms, notably the generation of reactive oxygen species (ROS) and nitric oxide (NO), thereby directly contributing to parasite elimination [34, 35]. In this context, P2X receptors function not only as modulators of inflammation but also as direct effectors of parasite killing.

Beyond its well-established role in innate immune cells, the purinergic receptor P2X7 has emerged as an important modulator of adaptive immunity, particularly through its effects on lymphocyte activation, differentiation, and survival [36]. P2X7 is functionally expressed in multiple T-cell subsets, including CD4⁺, CD8⁺, regulatory T cells (Tregs), and memory populations, where extracellular ATP acts as a danger-associated signal shaping immune responses [37–41]. Depending on the intensity and duration of stimulation, P2X7 activation can promote T-cell activation, cytokine production, and metabolic reprogramming, or alternatively induce cell death and contraction of specific lymphocyte subsets [42, 43]. Notably, P2X7 signaling has been implicated in the balance between effector and regulatory T cells, influencing Th1/Th17 polarization and Treg stability in inflammatory contexts [44]. In addition, P2X7 plays a critical role in the generation and maintenance of long-lived memory CD8⁺ T cells by regulating their metabolic fitness and responsiveness to extracellular cues [40]. Through these mechanisms, P2X7 serves as a key molecular link between inflammatory microenvironments and the fine-tuning of adaptive immune responses, contributing both to immune homeostasis and to the immunopathogenesis of infectious and inflammatory diseases [15].

Complementing the rapid ionotropic signaling mediated by P2X receptors, metabotropic P2Y receptors regulate longer-term adaptations in immune cell function and intracellular organization. Members of the P2Y receptor family orchestrate signaling programs that control immune cell migration, phagocytosis, cytokine production, and tissue repair [45–51]. Beyond immune modulation, P2Y receptor activation has been linked to alterations in vesicular trafficking, metabolic fluxes, and ionic gradients that can restrict intracellular parasite replication [52]. Their responsiveness to chemically diverse endogenous ligands, including nucleotides, dinucleotides, and nucleotide sugars, further broadens the pharmacological landscape for therapeutic targeting [45].

The contribution of purinergic signaling to infectious disease outcomes is now firmly supported by experimental evidence [12, 31, 53–56]. In parasitic infections, engagement of purinergic pathways influences parasite survival, replication, and dissemination while simultaneously shaping inflammatory responses and tissue pathology. Foundational studies in toxoplasmosis and malaria demonstrated that activation of P2 receptors can directly impair parasite viability, providing a conceptual framework for extending these observations to other intracellular parasitic diseases [57, 58].

Apicomplexan parasites, including *Toxoplasma gondii* and *Plasmodium* spp., establish intracellular niches characterized by intense host cell stress, metabolic reprogramming, and membrane remodeling (conditions that favor sustained extracellular nucleotide accumulation and robust P2 receptor activation) [59, 60]. Within this context, purinergic signaling emerges as a dual-action therapeutic axis, capable of directly targeting parasite viability while simultaneously modulating host immune responses. The following sections will therefore examine P2 receptors, particularly the P2X family, as promising therapeutic targets during *Toxoplasma gondii* and *Plasmodium* spp. infections.

## P2 receptors and toxoplasmosis

The protozoan *Toxoplasma gondii* is the causative agent of toxoplasmosis. This obligate intracellular parasite can infect nearly all nucleated cells of warm-blooded vertebrates and is estimated to affect about one-third of the world’s population [61]. In immunocompetent individuals, infection is generally asymptomatic or mild, progressing to a latent stage characterized by bradyzoite cysts that may persist chronically. In contrast, in immunosuppressed hosts, bradyzoites may convert into tachyzoites with high proliferative capacity, causing extensive tissue damage and parasite dissemination [62].

Following ingestion of infectious forms, either oocysts from feline feces or tissue cysts in undercooked meat, sporozoites or bradyzoites are released in the intestine, differentiate into tachyzoites, and disseminate through the bloodstream or infected leukocytes to multiple organs [63]. Tachyzoites replicate rapidly inside host cells, leading to lysis, necrosis, and mononuclear inflammatory infiltrates [64].

In immunocompetent hosts, this acute phase is typically subclinical or presents with mild, self-limiting symptoms such as fever, lymphadenopathy, and malaise [65]. The immune response, dominated by interferon-γ and T cells, restricts tachyzoite proliferation and drives their differentiation into bradyzoites, which form long-lasting tissue cysts mainly in the brain, heart, and skeletal muscles [66].

During chronic infection, pathology is minimal in healthy individuals. Under immunosuppression, however, latent bradyzoites may revert to tachyzoites, leading to renewed tissue destruction and inflammation [67]. In patients with AIDS, transplants, or chemotherapy, reactivation often results in toxoplasmic encephalitis, marked by necrotic foci, edema, and mixed inflammatory infiltrates, clinically manifesting as seizures, neurological deficits, and cognitive decline [68]. Disseminated disease may also compromise the lungs or myocardium [64].

Congenital toxoplasmosis develops when a primary maternal infection during pregnancy enables tachyzoites to traverse the placenta. Infection acquired in early gestation is associated with severe fetal damage, frequently resulting in intracranial calcifications, hydrocephalus, chorioretinitis, and long-term neurodevelopmental impairment [61].

The choice of therapy for toxoplasmosis is determined by the host’s immune status and clinical presentation. In immunocompetent individuals, infection is typically self-limiting and usually does not warrant treatment. For severe disease, congenital infection, or immunosuppressed patients, the recommended regimen combines pyrimethamine, sulfadiazine, and folinic acid. This protocol, however, is constrained by hematologic toxicity and poor tolerance [69]. When sulfadiazine cannot be used, clindamycin may serve as a substitute, while other agents such as trimethoprim-sulfamethoxazole, azithromycin, or atovaquone are considered secondary options depending on tolerance and availability [70].

During pregnancy, spiramycin is administered to minimize vertical transmission, but if fetal infection is confirmed, therapy must be shifted to pyrimethamine–sulfadiazine–folinic acid [71]. In infants with congenital toxoplasmosis, prolonged treatment with this combination has been associated with reduced parasite burden and better neurodevelopmental outcomes [72]. For immunocompromised patients, particularly those with HIV/AIDS or undergoing transplantation, the same regimen is employed, although prolonged therapy and secondary prophylaxis are required until immune function recovers [73, 74].

Despite these therapeutic strategies, currently available drugs cannot eradicate latent bradyzoite cysts. This limitation leads to frequent relapses and underscores the urgent need for novel treatments capable of targeting both tachyzoite and bradyzoite stages [75–77].

**Fig. 1:**
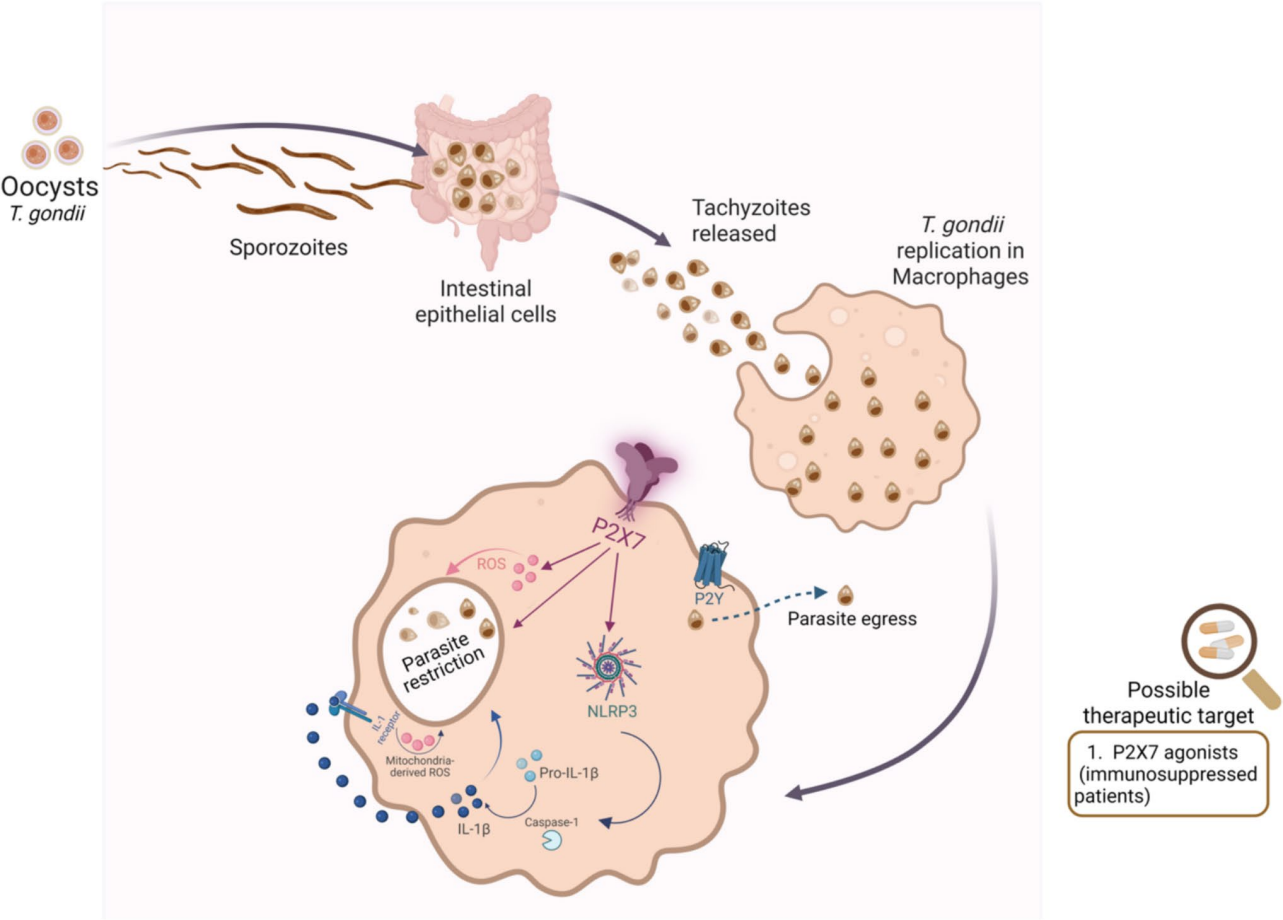
P2 receptor–mediated restriction of *Toxoplasma gondii* replication in macrophages. Following ingestion of *T. gondii* oocysts, sporozoites invade intestinal epithelial cells and differentiate into rapidly replicating tachyzoites, which subsequently infect macrophages. Activation of the P2X7 receptor in infected macrophages triggers antimicrobial pathways, including ROS production and NLRP3 inflammasome assembly, leading to caspase-1 activation and IL-1β maturation. Next, engagement of IL-1R triggers mitochondrial ROS production, ultimately leading to parasite death. Moreover, activation of P2Y receptors induces parasite egress, yielding a less virulent phenotype that is more readily targeted by host immune defenses. These coordinated responses contribute to intracellular parasite restriction and enhanced host resistance. Given its role in parasite control, P2 receptor agonism represents a potential therapeutic approach, particularly for immunosuppressed individuals at higher risk of severe toxoplasmosis. Created in BioRender. Carvalho, N. (2026) https://BioRender.com/b2y3a7s

The P2X7 receptor is a central component of host defense against *Toxoplasma gondii*. Its activation in macrophages [35, 78] and intestinal epithelial cells [79] induces antimicrobial effector mechanisms, including ROS production, NLRP3 inflammasome activation, and IL-1β release, thereby restricting parasite replication. Extracellular ATP–mediated stimulation of the P2X7 receptor limits *T. gondii* proliferation in macrophages by promoting NADPH oxidase–driven reactive oxygen species. Additionally, P2X7 activation triggers the canonical NLRP3 inflammasome pathway, leading to caspase-1 activation, increased IL-1β release, and amplification of mitochondrial ROS generation [80]. Accordingly, P2X7-deficient mice exhibit compromised immunity, showing increased susceptibility to acute infection with the type I RH strain and higher cyst burden and mortality during chronic type II ME-49 infection, associated with reduced IL-1β, IL-12, and ROS production (Fig. 1) [34, 81]. In cerebral toxoplasmosis, these mice succumb within 8 weeks, underscoring the essential role of P2X7 in resistance to brain infection. More recently, infection with atypical Brazilian strains, such as EGS, has confirmed this protective role: P2X7-deficient mice exhibit greater parasite dissemination and dysregulated inflammatory responses [82]. Consistently, loss of P2X7 exacerbates *T. gondii*-induced ileitis, leading to increased intestinal injury, parasite burden, hypercontractility, and liver damage, highlighting its role in coordinating inflammasome activity and host–microbiota interactions during infection [83]. In humans, genetic variation in *P2RX7* also appears to influence disease susceptibility: the nonsynonymous Gln460Arg variant was associated with increased risk of ocular toxoplasmosis in Colombian patients, suggesting that impaired P2X7 function may represent a biomarker of susceptibility [84]. Collectively, these findings support the notion that the P2X7 receptor is a promising therapeutic target for toxoplasmosis.

Stimulation with UTP or UDP triggers premature egress of *T. gondii* tachyzoites from infected macrophages in a Ca^2^⁺-dependent manner. Parasites forced to exit host cells early display markedly reduced infectivity during subsequent rounds of infection, failing to replicate efficiently or block lysosome–parasitophorous vacuole fusion (Fig. 1). Pharmacological studies using selective agonists and antagonists further indicate that this premature egress is mediated through activation of P2Y2, P2Y4, and P2Y6 receptors [57]. Thus, P2Y receptors may also represent promising targets for novel therapeutic strategies to control toxoplasmosis.

In conclusion, toxoplasmosis remains a major global health concern, particularly in regions with high circulation of virulent strains and among immunocompromised and pregnant individuals, where infection can cause severe neurological and ocular disease. In this context, purinergic signaling has emerged as a key host pathway controlling *T. gondii*. P2X7 activation triggers antimicrobial and inflammasome responses that restrict parasite replication and tissue damage, whereas stimulation of P2Y_2_, P2Y_4_, and P2Y_6_ promotes premature parasite egress and reduces subsequent infectivity. Taken together, these observations support the concept that P2 receptors constitute a strategically attractive, yet context-dependent, class of therapeutic targets that can enhance parasite control and improve outcomes in toxoplasmosis.

## P2 receptors and malaria

*Plasmodium *spp. are protozoan parasites responsible for malaria and are transmitted by hematophagous mosquitoes. Healthy individuals acquire the parasite during the blood meal of an infected Anopheles vector [85]. Infectious sporozoites, which display tropism for the liver, are inoculated during the bite and subsequently infect hepatocytes, initiating the pre-erythrocytic hepatic stage [86]. This stage is clinically silent, during which Plasmodium undergoes massive replication and generates merozoites. These merozoites then enter the bloodstream and invade erythrocytes, giving rise to the asexual blood stage responsible for the classic clinical manifestations of malaria [87].

Malaria is closely associated with environmental conditions and sociodemographic factors, including poverty [88, 89]. The primary causative agent is *P. falciparum*, which accounted for about 97% of global malaria cases in 2023 and is predominantly found in sub-Saharan Africa. This region was also responsible for 95% of malaria-related deaths in the same year [3]. *P. vivax* contributed to roughly 3.5% of cases and is prevalent in South America, South and Southeast Asia, the Western Pacific, and Oceania. Less common but widely distributed species include *P. malariae* and *P. ovale* [85, 90, 91].

The management of malaria predominantly relies on combination therapies designed to reduce the risk of drug resistance, which is suspected when parasite clearance from the bloodstream is delayed [92]. For uncomplicated P*. falciparum* malaria, where resistance to chloroquine is widespread, artemisinin-based combination therapies (ACTs) remain the cornerstone. These regimens pair a short-acting artemisinin derivative with a longer-acting partner drug such as lumefantrine or amodiaquine, with artemether–lumefantrine being the most commonly used formulation worldwide [85]. Other therapeutic options include alternative ACTs, atovaquone–proguanil, quinine, and mefloquine, though the latter is restricted by adverse neuropsychiatric effects. Chloroquine maintains utility only in areas where *P. falciparum* has remained sensitive [3].

*P. vivax* infections are generally responsive to chloroquine, although resistant strains are increasingly reported, particularly in regions such as Indonesia and Papua New Guinea, where ACTs have become the preferred option. Infections caused by *P. ovale*, *P. malariae*, and *P. knowlesi* may be treated with either chloroquine or ACTs, but ACTs are recommended in endemic areas with high resistance or when species identification is uncertain [3].

Cases of severe malaria require immediate treatment with intravenous artesunate, independent of the infecting species, followed by oral therapy once parasitemia declines and the patient can tolerate oral administration [88]. When intravenous access is not immediately available, temporary alternatives include oral or intramuscular antimalarials [89, 93]. Cerebral malaria, characterized by brain swelling, is associated with high mortality, and supportive or adjunctive treatments remain an active area of investigation [94]. Some studies suggest acetaminophen may confer renal protection, while interventions such as exchange transfusion have not shown clear clinical benefit [95, 96].

Although intravenous artesunate is highly effective, it has been associated in rare cases with delayed hemolysis, especially in patients with high parasite burdens [97, 98]. A more pressing concern is the emergence of artemisinin resistance, manifested by delayed parasite clearance. Initially observed in Southeast Asia, this resistance has now spread to multiple countries in East Africa, posing a serious challenge to malaria control efforts [99, 100].

Together, these developments underscore the urgent need for new antimalarial agents. The growing resistance to artemisinin and other drugs threatens the effectiveness of current therapies, making the discovery of innovative treatments essential to sustain global progress, reduce mortality, and maintain effective malaria control.

Malaria may present as asymptomatic parasitemia, mild illness, or severe disease. The classic symptoms of *Plasmodium* infection include chills, fever, and headache, and alterations in mental status may also occur [101]. Anemia is a common complication, particularly in young children and pregnant women, and in some endemic regions it accounts for more than 50% of malaria-related deaths [102]. Anemia may result from increased destruction of infected red blood cells [103].

Erythrocyte rupture during malaria results in substantial extracellular ATP release, which acts as a danger signal amplifying inflammatory cascades. ATP functions as a danger signal in inflammatory responses that can culminate in fever, one of the most characteristic symptoms of the disease [104, 105]. Activation of P2 receptors promotes the release of pyrogenic cytokines, which play a central role in the febrile response. By binding to purinergic receptors on the cell surface, ATP thus contributes to malaria pathogenesis [106].

Erythrocytes contribute to malaria pathology by adhering to endothelial cells within the cerebral microvasculature and other vital organs [107–109]. This adhesion can lead to neurological disturbances, including neurophagia and axonal injury [110–112]. These alterations result from factors such as hypoxia, hypoglycemia, cerebral edema, hemorrhage, and inflammation. As a consequence, cerebral malaria may induce cellular damage that stimulates the release of neurotransmitters, including ATP. Supporting this, Marín-García and colleagues demonstrated that *Plasmodium*-infected mice with neurological malaria exhibit altered purinergic receptor expression patterns across distinct brain regions [112].

**Fig. 2:**
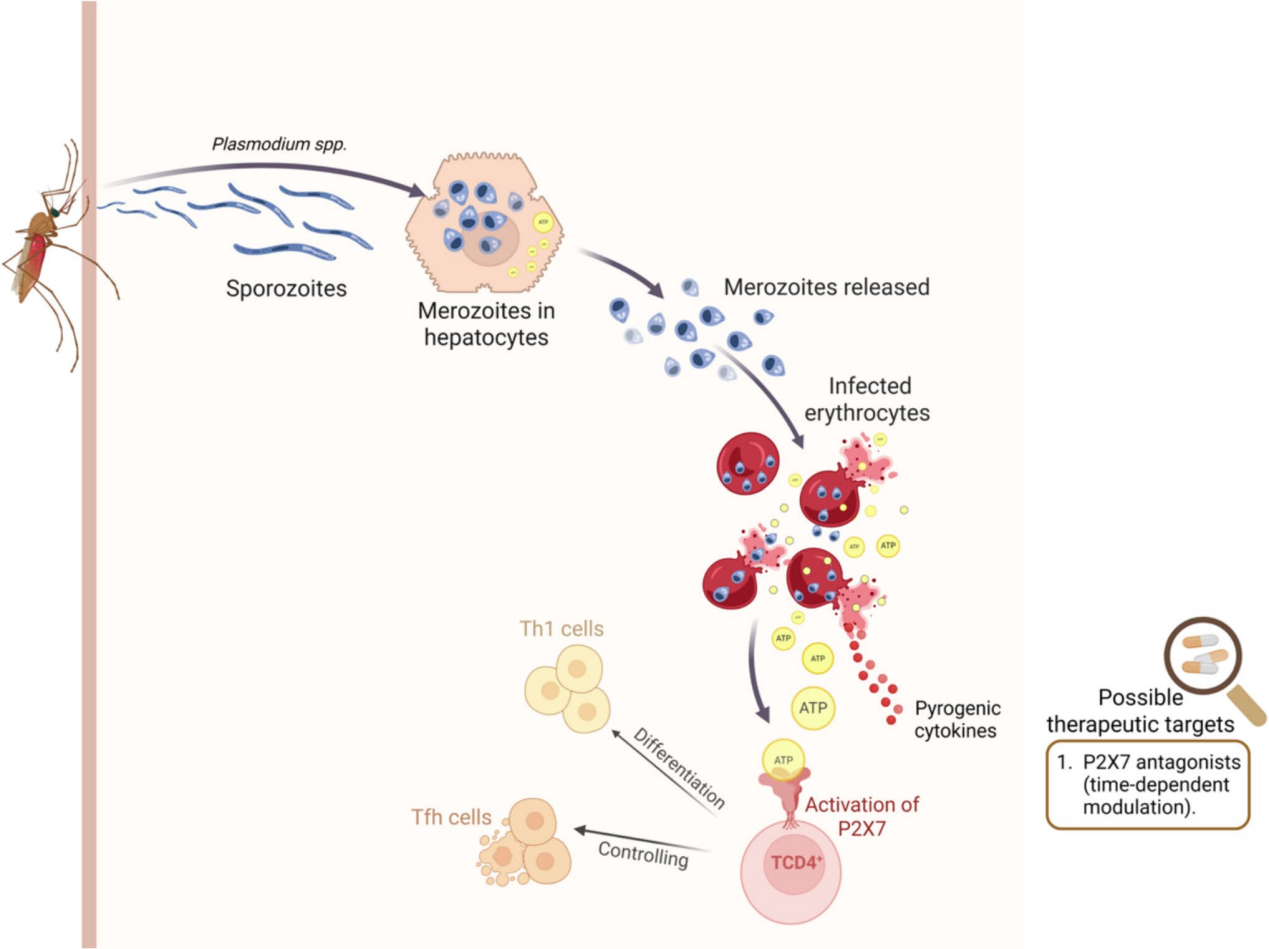
Role of P2X7 receptor signaling in immune modulation during *Plasmodium* infection. Following transmission by the mosquito vector, *Plasmodium* sporozoites migrate to the liver, where they replicate within hepatocytes and release merozoites into the bloodstream. Once inside erythrocytes, merozoite replication and hemolysis result in the release of ATP into the extracellular environment. Extracellular ATP can activate the P2X7 receptor, inducing pyrogenic cytokines that cause fever. On the other hand, P2X7 activation on CD4⁺ T cells influences their differentiation into Th1 or T follicular helper (Tfh) subsets, thereby shaping the adaptive immune response. P2X7-driven modulation of T cell responses may contribute to parasite control. The figure also highlights the therapeutic potential of time-dependent P2X7 antagonism to limit excessive inflammation while preserving protective immunity. Created in BioRender. Carvalho, N. (2026) https://BioRender.com/flvt962

Conversely, the P2X7 receptor is essential for T helper 1 (Th1) cell differentiation and for controlling the splenic follicular helper T (Tfh) cell population during blood-stage malaria, in part by promoting apoptotic-like cell death (Fig. 2) [58]. Salles et al*.* (2017) demonstrated that, upon rupture of infected erythrocytes, P2X7 is activated in CD4⁺ T cells, rendering them highly responsive to ATP. Mice deficient in P2X7 are more susceptible to infection due to impaired Th1 differentiation, and the receptor also restrains Tfh cell expansion. In addition, P2X7 signaling drives Th1 commitment by inducing metabolic reprogramming toward aerobic glycolysis [113]. Together, these findings highlight P2X7 as a critical regulator of Th1/Tfh balance and a key determinant of protective immunity during malaria.

In conclusion, malaria remains a major global health challenge due to its high prevalence, severe complications, and the emergence of resistance to frontline therapies. Beyond its clinical manifestations, disease pathogenesis is closely linked to host-parasite interactions that involve purinergic signaling, particularly through extracellular ATP and P2 receptors. While ATP release contributes to inflammation, fever, and neurological complications, P2X7 receptor activity plays a protective role by promoting Th1 differentiation and infection control. These findings highlight purinergic signaling pathways as both drivers of pathology and potential therapeutic targets, underscoring the importance of innovative approaches to improve malaria treatment and patient outcomes.

## Conclusion

The intricate interplay between apicomplexan parasites and their vertebrate hosts reflects a long evolutionary history marked by mutual adaptation and constraint. While this coevolution has limited opportunities for selectively targeting parasite metabolism, it has also revealed host-centered mechanisms that can be harnessed for therapeutic benefit. Purinergic signaling represents one such mechanism, translating infection-associated cellular stress into coordinated immune responses capable of restricting parasite growth. By sensing extracellular nucleotides released during host cell perturbation, P2 receptors occupy a strategic position at the interface between tissue damage, inflammation, and antimicrobial defense. The capacity of these receptors to influence both immune cell function and intracellular environments exploited by parasites underscores their relevance as modulators of disease outcome. A deeper understanding of how distinct P2 receptor subtypes contribute to host protection and pathology during apicomplexan infections may open new avenues for the development of innovative host-directed interventions that complement existing antiparasitic therapies.

## Data Availability

Do not apply.
